# CO-Releasing Molecules Have Nonheme Targets in Bacteria: Transcriptomic, Mathematical Modeling and Biochemical Analyses of CORM-3 [Ru(CO)_3_Cl(glycinate)] Actions on a Heme-Deficient Mutant of *Escherichia coli*

**DOI:** 10.1089/ars.2014.6151

**Published:** 2015-07-10

**Authors:** Jayne Louise Wilson, Lauren K. Wareham, Samantha McLean, Ronald Begg, Sarah Greaves, Brian E. Mann, Guido Sanguinetti, Robert K. Poole

**Affiliations:** ^1^ Department of Molecular Biology and Biotechnology, The University of Sheffield, Sheffield, United Kingdom.; ^2^ School of Informatics, The University of Edinburgh, Edinburgh, United Kingdom.; ^3^ Department of Chemistry, The University of Sheffield, Sheffield, United Kingdom.

## Abstract

***Aims:*** Carbon monoxide-releasing molecules (CORMs) are being developed with the ultimate goal of safely utilizing the therapeutic potential of CO clinically, including applications in antimicrobial therapy. Hemes are generally considered the prime targets of CO and CORMs, so we tested this hypothesis using heme-deficient bacteria, applying cellular, transcriptomic, and biochemical tools. ***Results:*** CORM-3 [Ru(CO)_3_Cl(glycinate)] readily penetrated *Escherichia coli hemA* bacteria and was inhibitory to these and *Lactococcus lactis,* even though they lack all detectable hemes. Transcriptomic analyses, coupled with mathematical modeling of transcription factor activities, revealed that the response to CORM-3 in *hemA* bacteria is multifaceted but characterized by markedly elevated expression of iron acquisition and utilization mechanisms, global stress responses, and zinc management processes. Cell membranes are disturbed by CORM-3. ***Innovation:*** This work has demonstrated for the first time that CORM-3 (and to a lesser extent its inactivated counterpart) has multiple cellular targets other than hemes. A full understanding of the actions of CORMs is vital to understand their toxic effects. ***Conclusion:*** This work has furthered our understanding of the key targets of CORM-3 in bacteria and raises the possibility that the widely reported antimicrobial effects cannot be attributed to classical biochemical targets of CO. This is a vital step in exploiting the potential, already demonstrated, for using optimized CORMs in antimicrobial therapy. *Antioxid. Redox Signal*. 23, 148–162.

## Introduction

Carbon monoxide (CO) is a gaseous signaling molecule in biology and medicine with numerous beneficial effects, including vasodilation, anti-inflammation, and anti-apoptosis (50). Carbon monoxide-releasing molecules (CORMs) (25, 44, 45) enable this noxious gas to be safely and selectively delivered, and they have also been exploited as antibacterial agents (15–17, 48, 52, 68, 69). However, CO gas is surprisingly ineffective in inhibiting microbial growth. Thus, bacteria have been grown with high concentrations of CO to maximize expression of heterologous globins (62) and CO is much less effective than CORMs in inhibiting bacterial growth (52) and respiration (74). It is tacitly assumed that the oxygen-binding hemes of globins and oxidases are prime targets of CO, CORMs, and other gasotransmitters, such as NO and H_2_S.

However, recent studies on the interactions of CORMs with bacteria suggest that these compounds have numerous targets and that toxicity is the outcome of diverse effects, many of which cannot be traced directly to the actions of CO (17, 48, 74). Indeed, the literature contains several reports of nonheme targets of CO (31), including examples of nonheme iron(II) carbonyls, including metals coordinated to S from cysteine and/or N from histidine. These ligands may constitute targets in cation channels (21) or in bacterial ion channels, leading to subsequent respiratory stimulation (74). Other examples of nonheme interactions include CO binding to iron in [Fe]-, [Fe-Fe]- and [Fe-Ni]-hydrogenases as in *Chlamydomonas* (66). CO also binds to binuclear copper sites, for example in tyrosinase (38) and hemocyanins (22). In CO dehydrogenase, which oxidizes CO to CO_2_, CO interacts with the nickel ion in one of the metalloclusters (“C-cluster”) (39). Endogenous CO is regarded as an important factor in natural redox signaling (7) and promotes a prooxidant milieu in aerobic mammalian cells (55). CORMs too may generate oxidative stress (51, 67).

Innovation Although carbon monoxide (CO) gas has therapeutic benefits, and carbon monoxide-releasing molecules (CORMs) are promising antimicrobial agents, their biological targets are poorly understood. By using bacteria that lack all hemes, this work provides the most direct evidence to date that CORMs target other cellular processes. CORM-3, and to a lesser extent its CO-depleted form, has comprehensive time-dependent effects on transcript profiles and transcription factor activities in a heme-deficient mutant of *Escherichia coli*. Particularly affected are iron acquisition, membrane stress resistance, and zinc transport. Our work highlights the need for integration of chemistry, physiology, and molecular biology before CORMs are clinically used.

Here, we exploited the ability to study in *Escherichia coli* and *Lactococcus lactis* the effects of a CORM in cells totally devoid of heme proteins. We tested the effects of CO administered *via* CORM-3 [Ru(CO)_3_Cl(glycinate)], the first water-soluble metal-based CORM (11, 33), and the focus of many detailed physiological and biochemical studies. To elucidate the mechanisms of CORM-3 activity in heme-deficient *E. coli*, we assessed gene expression changes of a *hemA* mutant and the isogenic wild-type strain *via* transcriptome profiling and statistical data modeling, together with protein quantitation and studies of membrane integrity. Importantly, analyses were also performed on mutant cells exposed to inactive CORM-3 (*i.e*., CORM-3 depleted of CO) (11, 41, 48) to address the critical question: are the observed effects CO specific?

## Results

### CORM-3 is bactericidal against heme-deficient bacteria

The *hemA* (=*hemM*) gene of *E. coli* encodes a glutamyl-tRNA reductase that catalyzes the second step in heme biosynthesis, leading to glutamate 1-semialdehyde (5). The next intermediate is δ-aminolevulinic acid (δ-ALA), which limits heme biosynthesis (71). Strains of *E. coli* carrying a *hemA* mutant allele (28, 30) cannot grow on oxidisable substrates when δ-ALA is not present, a phenotype attributed to the absence of functional cytochromes, and grow only fermentatively. Reconstitution of oxidase activity and formation of a functional proton-translocating respiratory chain (28–30) can be achieved by incorporation of heme into pre-existing apoproteins.

**FIG. 1.**
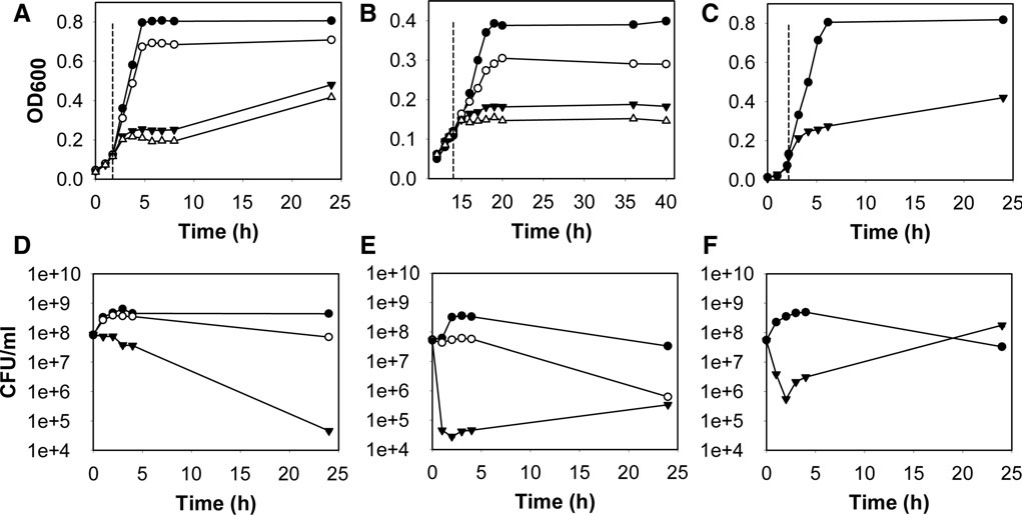
**Heme-deficient** ***Escherichia coli*** **are more CORM-sensitive than isogenic wild-type or heme-reconstituted bacteria.** Cultures were grown anaerobically and stressed with 100 μ*M* (*open circles*), 200 μ*M* (*closed triangles*), and for growth studies only, 300 μ*M* (*open triangles*) CORM-3 at an OD_600_ of ∼0.1 (*dashed line*). Growth and viability of CORM-3-treated cultures were compared with control cultures (nothing added, *closed circles*). To determine the effects of CORM-3 on growth, hourly OD_600_ readings were taken for 6 h post-addition of the compound, followed by a final 24 h reading. Wild type **(A)**, heme-deficient mutant **(B),** and cells reconstituted for heme **(C)** by adding δ-ALA (0.1 m*M* final concentration). For viability assays, a sample was taken immediately before addition of the compound, followed by sampling every hour for 4 h post-stress and at 24 h to complete the experiment. Wild type **(D)**, heme-deficient mutant **(E),** and cells reconstituted for heme **(F)** by adding δ-ALA. Data show patterns seen in ≥3 biological replicates. Viability data are plotted as means±SEM from ≥3 individual spots. Note that the scale on the y-axis is logarithmic in base 10, hence 1e+3=1000.

We first verified that the *hemA* strain constructed by P1 transduction lacked cytochromes (Supplementary Fig. S1A; Supplementary Data are available online at www.liebertpub.com/ars) and was unable to grow on nonfermentable substrates, such as glycerol or succinate (not shown). Cultures of the heme-deficient mutant and wild type strains were then stressed with CORM-3 or inactive CORM-3 (iCORM-3). Micromolar concentrations of CORM-3 resulted in a concentration-dependent slowing of growth (Fig. 1A–C) for the wild-type strain, *hemA* mutant, and the mutant after reconstitution with δ-ALA. Wild-type cultures stressed with 100 μ*M* CORM-3 showed a marginally increased doubling time (0.81±0.18 h) compared with the control (0.79±0.13 h) but, at 200 and 300 μ*M* CORM-3, growth was prevented for 5 h. At these concentrations, cultures showed some recovery between 8–24 h, but cell densities did not reach the level of control or 100 μ*M*-treated cultures (Fig. 1A). Similar results were obtained for the *hemA* mutant; 100 μ*M* CORM-3 increased the doubling time from 2.0±0.33 h (control) to 3.6±1.3 h. Unlike the control, mutant cultures did not recover even after 28 h of incubation with the CORM (Fig. 1B).

It should be noted that supplementation with δ-ALA restored growth yields to the *hemA* mutant (compare Fig. 1A, C) but did not fully restore cell viability, as the number of viable cells declined after a few hours (compare Fig. 1D, F). The reason is unclear, but it may reflect a drain on ATP required for cytochrome biosynthesis from δ-ALA (30). The δ-ALA-reconstituted mutant (Fig. 1C) was also inhibited by 200 μ*M* CORM-3, with the doubling time increasing from 0.86±0.14 h to 2.9±1.0 h. Interestingly, growth of the wild-type strain was significantly slowed (doubling time increasing typically from 1.0 h to 1.75 h) by CO gas bubbled into cultures at 100 ml min^−1^ (Supplementary Fig. S2A), but growth of the heme-deficient mutant was unaffected (Supplementary Fig. S2B). The reduction in growth rate of wild-type cultures by CORM-3 may be due to a metabolic switch from anaerobic respiration, where CO binds to hemes of the anaerobic respiratory chain, to mixed-acid heme-independent fermentation, which supports lower growth rates.

iCORM-3 was prepared by a standard procedure to give a preparation that releases to ferrous myoglobin typically <5% of the CO delivered by equimolar CORM-3. iCORM-3 had a concentration-dependent effect on the mutant (Supplementary Fig. S3B), increasing the doubling time from 1.76±0.19 h (control) to 2.0±0.25 h (CORM-3) but not to the same extent as CORM-3 (Fig. 1). Inactive CORM-3 (iCORM-3, 200 μ*M*) also marginally increased the doubling time of the wild-type strain (from 0.76±0.14 h, control; 0.80±0.09 h, 200 μ*M* CORM-3), but the effect was insignificant when compared with CORM-3 (Supplementary Fig. S3A). This minor growth inhibition may be explained by residual CO release (Supplementary Fig. S3A inset).

Viability assays revealed a gradual decline in wild-type cell counts after addition of 200 μ*M* CORM-3 by 0.5-log over 3–4 h, with a 2- to 3-log drop within 24 h (Fig. 1D). However, mutant cell viability dropped by more than 3-log within the first hour of CORM-3 treatment (Fig. 1E). On adding 100 μ*M* CORM-3, wild-type cell counts were similar to those of the control culture for 4 h, with a 0.5-log drop within 24 h (Fig. 1D), and levels of viable mutant cells remained static for 4 h, but dropped by 2-log within 24 h (Fig. 1E). Viability of both strains was unaffected for 4 h post-addition of iCORM-3 (Supplementary Fig. S3). However, after 24 h, the viability of wild-type cultures treated with 100 and 200 μ*M* was reduced by 1- and 3-log, respectively (Supplementary Fig. S3C). At 24 h, 200 μ*M* iCORM-3 decreased the mutant cell count by 2-log, while 100 μ*M* was ineffective (Supplementary Fig. S3D). Surprisingly, although control δ-ALA-supplemented cells showed declining viability (Fig. 1F), those treated with CORM-3 showed a recovery after an initial precipitous drop (Fig. 1F). The explanation is unclear but Haddock (30) calculated that even 10 μ*M* δ-ALA (10% of the concentration used here) is adequate for the synthesis of a 10-fold higher cytochrome concentration than that found in reconstituted cells. We speculate that excess δ-ALA, or hemes synthesized in its presence that are not dithionite reducible (Fig. S1), may bind CORM-3, which is known to bind, for example, to exposed histidine residues (10).

To examine whether CORM-sensitive growth is a general feature of heme-deficient bacteria, we also studied *L. lactis* (Supplementary Fig. S4A, B), a Gram-positive, homo-fermentative bacterium that is naturally devoid of heme, yet whose capacity for respiration can be invoked by growth with exogenous heme (19). At 200 μ*M*, CORM-3 completely suspended growth within 0.5 h and caused a gradual decrease in viability, with a 2.5- to 3-log drop after 24 h (Supplementary Fig. S4A, C). At 100 μ*M*, the compound allowed continued growth for 1 h, but then suspended growth, decreased the stationary phase population by ∼15%, and reduced cell viability by 0.5- to 1-log over 24 h (Supplementary Fig. S4A, C). In contrast, iCORM-3, even at 200 μ*M*, barely inhibited growth of *L. lactis* and was without measurable effect on viability (Supplementary Fig. S4B, D).

Thus, CORM-3 is a potent bactericidal agent, even against heme-deficient bacteria that do not respire. Thus, CORM or CO cannot be responsible for generating oxidative stress by blocking respiratory electron transfer and promoting superoxide formation (49). The data comparing CO gas and iCORM-3 suggest that the ruthenium co-ligand fragment (iCORM-3) together with the labile CO produce a synergistic effect that is important for the full toxicity of CORM-3.

### Reconstitution of the heme-deficient mutant with δ-ALA reduces CORM-3 toxicity

To determine whether reconstitution of heme protects against CORM-3, we exposed cultures of the heme-deficient mutant, grown in the presence of δ-ALA, to the compound. Reconstitution was confirmed in spectra that revealed characteristic heme signals (Supplementary Fig. S1A, B) (30). After reconstitution of cellular hemes *in vivo*, the mutant control culture (no CORM-3) reached a maximum optical density (OD) (Fig. 1C) that was comparable with wild-type controls (Fig. 1A). Although growth was slowed within 1 h after exposure to the compound, the kinetics were similar to those seen for the wild-type (compare Fig. 1A, C) but not the cytochrome-deficient *hemA* mutant (compare Fig. 1B, C). Over the remaining 20 h, cells reconstituted with δ-ALA grew slowly (Fig. 1C), mimicking growth of the wild-type strain under these conditions (Fig. 1A) but unlike the *hemA* mutant (Fig. 1B). After reconstitution with δ-ALA, the untreated culture showed an ∼1-log increase in the number of viable cells, followed by a slow decline (Fig. 1F). In contrast, the culture treated with 200 μ*M* CORM-3 showed a precipitous 2-log drop in viable cells over the first 2 h after addition of CORM-3 (Fig. 1F) and cell numbers then recovered. Thus, reconstitution with δ-ALA restores growth, measured as culture OD, to levels seen in the wild-type strain at 200 μ*M* CORM-3, and cultures are protected from the 3-log CORM-induced drop in viability that is seen in the mutant (compare Fig. 1E, F). Thus, respiration in the presence of CO is not detrimental to growth as a result of reactive oxygen species generation.

### Cellular retention of CO is reduced in the heme-deficient mutant of *E. coli*

**FIG. 2.**
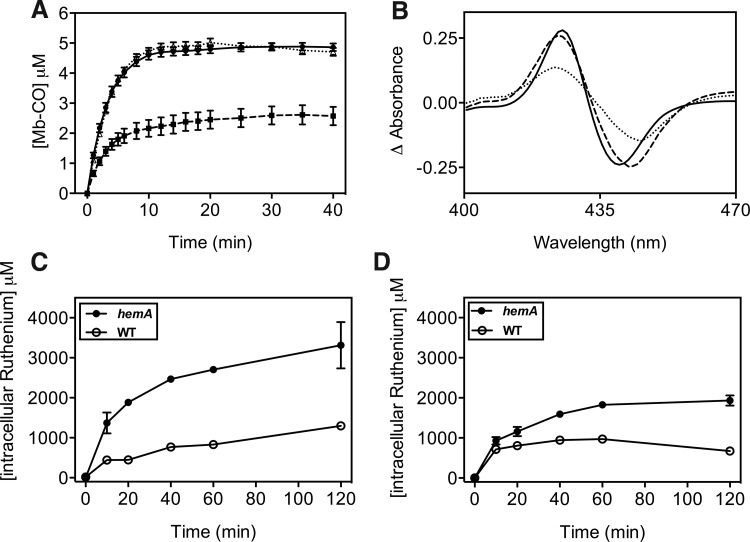
**CO retention and access of CORM-3 to the cell interior is dependent on heme. (A)** Myoglobin (10 μ*M*) and CORM-3 (8 μ*M*) were added to buffer only (*circles*), wild-type (*squares*), or *hemA* mutant (*triangles*) cells in the presence of Na dithionite. The concentration of Mb-CO accumulated was measured at several time points in CO difference spectra. Data are plotted as means±SEM from ≥5 replicates. **(B)** CO-reduced *minus* reduced spectra of myoglobin (10 μ*M*) and CORM-3 (8 μ*M*) added to buffer only (*solid line*), wild-type (*dotted line*), or *hemA* mutant (*dashed line*) cells in the presence of Na dithionite at *t*=5 min. **(C, D)** Intracellular ruthenium levels in *hemA* (*closed circles*) and wild-type (*open circles*) cells were measured by ICP-AES over 120 min after exposure of cultures to 100 μ*M* CORM-3 under anaerobic **(C)** or aerobic **(D)** conditions. Data are plotted as means±SEM from ≥3 biological replicates.

To assess CO removal from CORM-3 and intracellular binding of CO in wild-type and heme-deficient *E. coli*, we followed formation of extracellular carboxymyoglobin (Mb-CO) over time after adding CORM-3 to bacterial suspensions in buffer in the presence of exogenously added myoglobin (Fig. 2A, B). Myoglobin cannot enter cells and so acts as a “sink” for unbound CO that would otherwise freely diffuse through membranes. Wild-type cells retained a significant amount of the CO released from CORM-3 (∼50%), making it unavailable to the extracellular myoglobin (Fig. 2B). However, in the heme-deficient strain, the accumulation of Mb-CO mirrored the pattern observed after addition of CORM-3 to myoglobin in buffer alone. That is, CO is not retained by *hemA* bacteria that lack a “CO trap.”

To determine whether this retention was due to CORM transport to the cell interior, we measured intracellular ruthenium levels after adding CORM-3 to cultures. Under anaerobic conditions (as in Fig. 1), *hemA* mutant cells accumulated ruthenium to levels that are three-fold higher than wild-type cells (Fig. 2C). Aerobically (Fig. 2D), the ruthenium concentration in *hemA* cells was two-fold higher than in the wild-type strain. Thus, CORM-3 enters bacteria *via* a heme-independent mechanism and accumulates to a greater degree in the absence of heme, perhaps as a result of impaired CORM efflux.

### CORM-3 and iCORM-3 elicit multifaceted transcriptomic effects even in the absence of heme proteins

The differences in gene expression between the mutant and wild-type under control conditions, that is, before adding CORM-3, are shown in Supplementary Fig. S5. Only 6% of the genome in the mutant (of a total of 4,598 genes) were significantly altered (summing the percentages changing up or down) in comparison with the wild type (Fig. S5A). Furthermore, the changes in expression were small, with only 0.6% of the genome being altered by ≥10-fold (Fig. S5B). As expected for a mutation affecting primarily respiratory metabolism, the genes that changed most in the *hemA* strain are implicated in iron-sulfur (Fe-S) protein assembly and metabolism, energy metabolism, glycolysis, the TCA cycle and fermentation, and membrane transport.

**FIG. 3.**
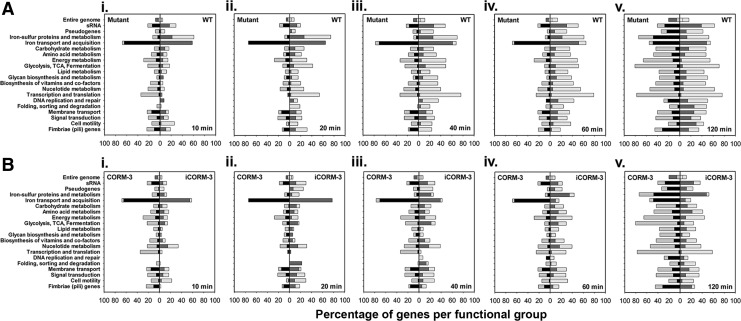
**Functional categories of genes affected by CORM-3 in the heme-deficient mutant and wild-type strains and a comparison of gene changes in the** ***hemA*** **mutant treated with CORM-3** ***versus*** **iCORM-3.** Cultures were grown anaerobically in defined medium. The bars show the percentage of genes in each group that exhibit altered expression after treatment with 100 μ*M* CORM-3. **(A)** Data are shown for the *hemA* mutant (*left* of the *midline* in each panel) and the wild-type (WT, *right* of the *midline* in each panel). Data are shown for cells at 10 min (**i**), 20 min (**ii**), 40 min (**iii**), 60 min (**iv**), and 120 min (**v**) after addition of CORM-3. **(B)** The bars show the percentage of genes in each group that exhibit altered expression after treatment with 100 μ*M* CORM-3 (*left* of the *midline* in each panel) and iCORM-3 (*right* of the *midline* in each panel) in the *hemA* mutant. Data are shown for cells at 10 min (**i**), 20 min (**ii**), 40 min (**iii**), 60 min (**iv**), and 120 min (**v**) after addition of CORM-3. For each group of data (WT *vs*. *hemA or* CORM-3 *vs*. iCORM-3), the *darker bars* in each category indicate the percentage of upregulated genes and the paler bars indicate the percentage of downregulated genes.

To provide an in-depth, time-resolved assessment of the response of the heme-deficient mutant and wild-type strains to CORM-3 and iCORM-3, we performed transcriptomic analyses, sampling cultures after CORM-3 addition to both wild-type and *hemA* mutant cultures. The CORM-3 added (100 μ*M*) was sufficient to challenge cells without significantly reducing viability within the timeframe of the experiment (Fig. 1D, E). The genome-wide effects of CORM-3 are revealed for each sampling point by the percentages of up- and downregulated genes in a number of functional categories (Fig. 3). Based on the proportions of genes in each class, the wild-type initially (20–60 min after CORM-3 addition) responds to CORM-3 more than the mutant but, by 120 min, the responses are similar (Fig. 3A). The upregulation at 10–60 min in both strains of genes involved in iron transport and acquisition is striking. However, at 20–120 min after CORM-3 addition, genes in most functional classes are down-, not up-, regulated in both strains (Fig. 3A).

We estimated the impact of CORM-3 *versus* iCORM-3 on the entire genome by measuring the percentages of genes changing either up or down (Fig. 3B). After 120 min of exposure to CORM-3, 5.7% of the genome was upregulated in the *hemA* mutant, and 14.4% was downregulated. For iCORM-3, these percentages were lower: 4.8% of the genome was upregulated and 6.6% was downregulated. Importantly, the fractions of the genome changing after 120 min in the wild-type strain were 5.4% up and 11% down. Thus, the *hemA* mutant experiences the impact of CORM-3 to a greater degree than the wild-type strain: summing up the up- and downregulated genes at 120 min for the *hemA* mutant, the value is 20%, whereas for the wild type strain the value is 16.3%.

### Genes involved in iron transport and acquisition are highly upregulated in response to CORM-3 in the heme-deficient mutant

**FIG. 4.**
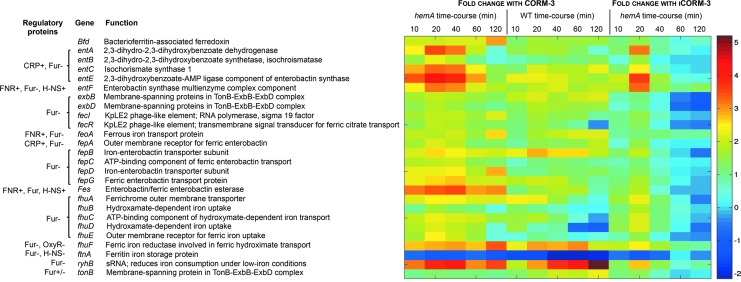
**Differential expression of genes involved in iron transport and acquisition.** The heat map quantifies the changes elicited by CORM-3 and iCORM3 in selected genes; it should be noted that the “heat scale” at the *right* is expressed as the natural logarithm of the fold changes in individual genes of the heme-deficient mutant of *E. coli* (*hemA*) and the corresponding wild-type grown anaerobically in defined medium after addition of 100 μ*M* CORM-3 or, for the mutant only, 100 μ*M* iCORM-3.

The category of genes most affected by CORM-3 were those encoding iron transport and acquisition functions; even after 10 min of exposure, ∼60% of such genes increased in expression in both the *hemA* and wild-type strains (Fig. 3A). The heat map in Figure 4 quantifies the changes elicited by CORM-3 and iCORM3 in selected genes involved in iron acquisition; it should be noted that the “heat scale” at the right is expressed as the natural logarithm of the fold change. Genes involved in the biosynthesis of the catecholate siderophore enterobactin (*ent*) were the most highly altered, with expression levels reaching 80-fold in the mutant and 10-fold in the wild-type strain (Fig. 4). Upregulated genes across all conditions tested also included the following: (i) *fepA*, which encodes an outer membrane (OM) protein that actively transports ferric enterobactin into the periplasm; (ii) *fepBCDG*, which encodes an ABC transporter that transports the iron(III)-bound siderophore through the cytoplasmic membrane (9); and (iii) *fes*, which encodes an enterobactin/ferric enterobactin esterase for intracellular breakdown of the ferrated carrier (8). The *fes* gene was also more highly upregulated in the mutant (19–35-fold) compared with the wild-type (2–6-fold) in response to CORM-3. Genes encoding the hydroxamate siderophore uptake system (*fhu*) that enables utilization of ferrichrome, ferric coprogen, and ferrioxamine B as sources of iron under low iron conditions were upregulated, as were genes encoding the ferric citrate (*fec*) and ferrous iron-uptake (*feoA*) systems (Fig. 4). Overall, the transcriptomic analysis reveals a marked enhancement of the expression of genes involved in iron scavenging in the *hemA* mutant when treated with CORM-3.

Genes involved in iron homeostasis were also affected by CORM-3 (Fig. 4). The gene encoding the iron storage protein, ferritin (*ftnA*), was downregulated in both the mutant and the wild-type (2–4-fold in the mutant, and 5–8-fold in the wild-type). In contrast, *bfd* (encoding bacterioferritin-associated ferredoxin) was upregulated (8–20-fold in the mutant, and 3–6-fold in the wild-type). It has been suggested that Bfd is involved in the release or delivery of iron to/from bacterioferritin, or other iron complexes (2). Interestingly, an sRNA gene, *ryhB*, was highly upregulated in both the mutant (21–62-fold) and the wild-type (7–181-fold). RyhB reduces iron consumption under low-iron conditions by downregulating iron-containing proteins, including ferritins, superoxide dismutase, and some genes of the TCA cycle (47), as well as promoting enterobactin synthesis (64). Collectively, the data point to iron deprivation or the perception of iron starvation in the *hemA* mutant when challenged with CORM-3.

**FIG. 5.**
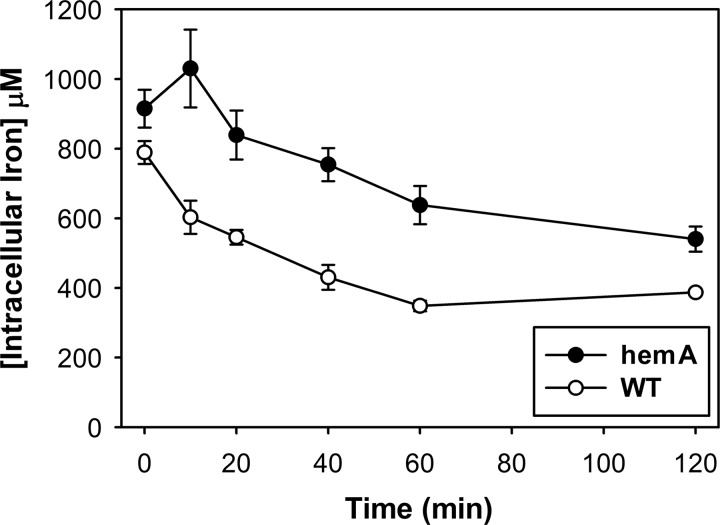
**CORM-3 treatment depletes iron levels in wild-type and heme-deficient** ***E. coli*** **cells over time.** Intracellular iron levels in *hemA* (*closed circles*) and wild-type (*open circles*) cells were measured by ICP-AES over a time-course of 120 min after exposure of cultures to 100 μ*M* CORM-3. Data are plotted as means±SEM from ≥3 biological replicates.

Since transcript levels for genes involved in iron homeostasis were altered, suggesting a shortage of biologically available iron induced by CORM-3 stress, we measured intracellular iron levels in wild-type and *hemA* cells under the same conditions used for microarray experiments. The level of iron was higher in the heme-deficient mutant at all time-points; however, after CORM-3 addition, iron levels dropped by ∼50% in both cultures over 120 min (Fig. 5). A *hemA* mutant *of Salmonella enterica* also showed marginally higher free iron levels than the wild-type strain (20).

### CORM-3 differentially alters transcription of genes involved in iron-sulfur cluster assembly and repair

Genes implicated in processes involving Fe-S proteins were differentially altered in the two strains as revealed by heat plots (Supplementary Fig. S6). Generally, genes encoding the housekeeping Fe-S cluster assembly system (*isc*) were downregulated in the wild-type and upregulated in the *hemA* mutant, irrespective of whether CORM-3 or iCORM-3 was used. In contrast, the *suf* genes involved in building Fe-S clusters during iron starvation and oxidative stress (18, 53, 63) were upregulated under most conditions, perhaps consistent with the slight loss of iron during CORM-3 treatment (Fig. 5). Finally, *ytfE* was upregulated in the mutant (≤18-fold) and unaffected in the wild-type, irrespective of whether CORM-3 or iCORM-3 was used (Supplementary Fig. S6). The product of this gene has been implicated in the repair of damaged Fe-S clusters, and its expression is stimulated by iron starvation (35, 54).

### CORM-3 perturbs the expression of genes involved in general stress response, zinc homeostasis, and signal transduction

**FIG. 6.**
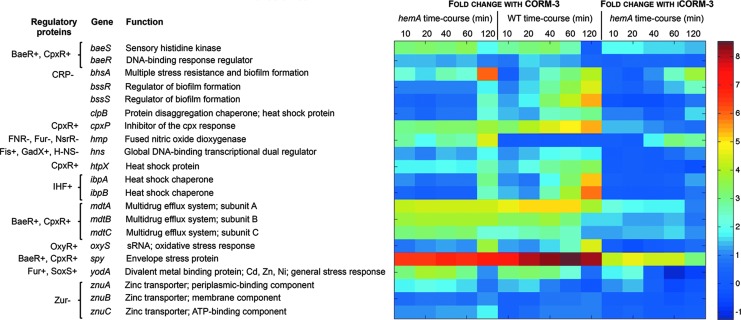
**Differential expression of genes involved in general stress responses, signal transduction, and zinc homeostasis.** The heat map quantifies the changes elicited in selected genes; the “heat scale” at the *right* is expressed as the natural logarithm of the fold change. Shown are individual genes of the heme-deficient mutant of *E. coli* (*hemA*) and the corresponding wild-type grown anaerobically in defined medium after addition of 100 μ*M* CORM-3 or, for the mutant only, 100 μ*M* iCORM-3.

Some of the most highly altered genes in this study are involved in signal transduction and general stress response. Genes shown in Figure 6 were the most altered, and many have been reported to change in response to CORM-3 stress in previous transcriptomic studies (15, 51). Exceptions include the upregulation of *hns*, whose product is a global transcriptional regulator that responds to environmental changes and stress, and marked upregulation in the mutant of *hmp*, encoding a flavohemoglobin (70) with NO dioxygenase activity (26).

The most upregulated gene in this study was *spy*, as previously reported (15, 48), which exhibited a 600–1000-fold change in the mutant and a 1200–5100-fold change in the wild-type. Upregulation was also noted in the mutant treated with iCORM-3, but by ≤10% of that seen after stressing with CORM-3 (21-109-fold), reflecting the reduced ability of iCORM-3 to release CO. Other genes within the same regulatory network as *spy* (*i.e*., regulated by BaeR and/or CpxR) were also greatly altered, including *mdtABC* encoding the multidrug efflux system, as well as *baeR* and *cpxP*. In addition, consistent with previous studies (15, 51), genes encoding proteins that respond to intracellular stresses and biofilm formation were upregulated to varying extents: *bhsA*, *bssR*, *bssS*, *clpB*, *htpX*, *yodA*, *ibpA*, and *ibpB*. Collectively, these patterns point to a profound stress at the cell membrane after exposure of the mutant and the wild-type to CORM-3 and, to a lesser extent, iCORM-3. However, expression levels of the majority of the genes were much higher in the wild-type than in the mutant.

Some of these genes also have roles in zinc homeostasis, namely *spy* and *yodA*, as well as the *mdtABC* operon that is upregulated in response to zinc (40). Furthermore, genes encoding the zinc(II)-transporter protein (*znuABC*) were upregulated, particularly in the mutant treated with CORM-3 (Fig. 6). The compound may therefore elicit a general effect on metal homeostasis.

### CORM-3 induces the expression of Spy protein and downregulates the production of CpxP, a regulatory protein involved in the cell stress response

**FIG. 7.**
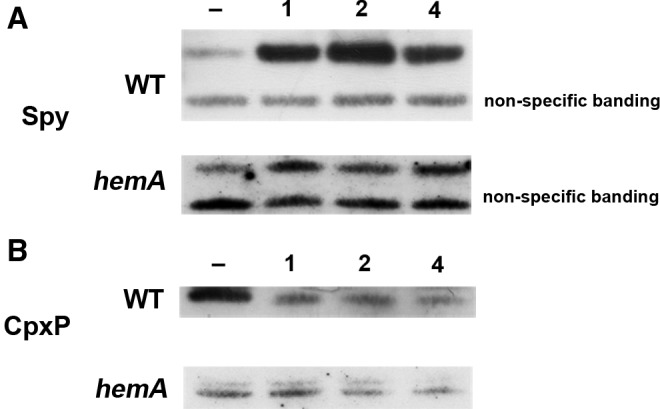
**Levels of Spy and CpxP protein are altered in response to CORM-3 in wild-type and** ***hemA*** **cells.** Western blotting of subcellular fractions was carried out in the absence and presence of 20 μ*M* CORM-3. **(A)** A typical Western blot is shown in the absence of CORM-3 (lane 1), or with 1, 2, or 4 h of incubation for wild-type or *hemA* cells with anti-Spy and **(B)** anti-CpxP. Bands of nonspecific binding of antibody are shown to demonstrate equal loading of protein in each lane.

It is clear that upregulation of *spy* at the transcriptomic level, along with transcripts such as *cpxP,* is indicative of the upregulation of the Cpx, or “cell envelope stress,” response when cells are exposed to CORM-3. Although these effects at the transcriptomic level are marked, it was important to determine whether the rise in level of transcripts reflects physiological production of protein. Western blot assays were therefore carried out using antisera to two key players in the response: Spy and CpxP. Spy was detected in periplasmic fractions of wild-type cells as expected. However, it was only readily detected in soluble, presumably cytoplasm-derived, fractions of *hemA* cells; the reason is unclear. For immunoblotting with CpxP, soluble fractions were used for *hemA* and the isogenic wild-type strain. Loading controls confirmed equal loading of protein in each well (Coomassie-stained gels, not shown), and nonspecific binding in the case of the Spy blots also indicate equal sample loading. As shown in Figure 7A, addition of only 20 μ*M* CORM-3 leads to a large increase in cellular protein after 1 h of incubation of the compound in wild-type cells; further incubation did not significantly increase Spy levels. The control in the absence of CORM shows very little Spy protein. Interestingly, the level of Spy protein is also increased with time after addition of CORM to *hemA* cells.

In addition to Spy, levels of the periplasmic chaperone CpxP were also determined. As shown in Figure 7B, levels of CpxP are higher in wild-type cells in the absence of CORM-3. A slight decrease in CpxP levels at 4 h compared with that at 1 h after CORM treatment showed a further slow decline in CpxP abundance. This is expected, since CpxP is a negative regulator of the Cpx response (60); if the Cpx response is to be active, levels of CpxP must be minimal. The result is also confirmed in *hemA* cells, with lower levels of CpxP protein after 4 h of incubation with CORM-3. These results, taken together with high levels of Spy protein, suggest that the induction of the Cpx response is a global consequence of CORM addition to cells.

### Membrane perturbation by CORM-3

**FIG. 8.**
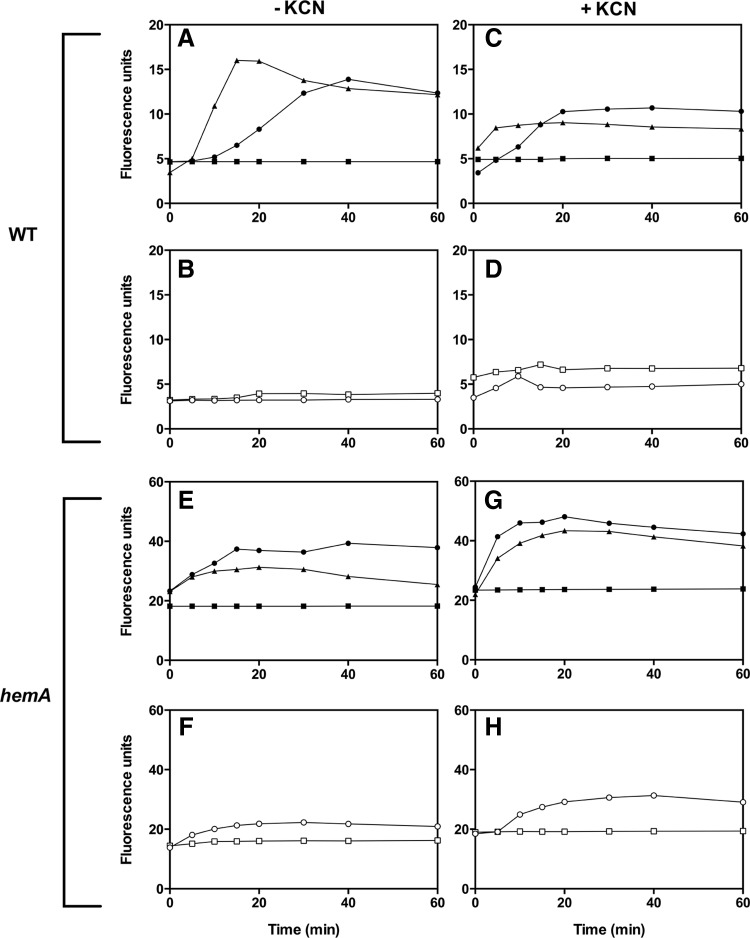
**CORM-3 perturbs the outer membrane of** ***E. coli*** **in both wild-type and** ***hemA*** **cells.** Wild-type **(A**–**D)** and *hemA* cells **(E**–**H)** were washed, resuspended in PBS, adjusted to an OD_600_ of ∼0.5, and then exposed to NPN alone (*squares*), NPN+30 μ*M* CORM-3 (*circles*), or NPN+100 μ*M* CORM-3 (*triangles*) **(A**, **C**, **E**, **G)**. Data are shown for measurements in the absence (*left*) or presence (*right*) of cyanide (KCN). In control experiments **(B**, **D**, **F**, **H)**, the compounds used were 100 μ*M* iCORM-3 (*open circles*) or 100 μ*M* CO gas in solution (*open squares*). All concentrations given are final concentrations in the fluorescence cuvette. Data are representative of ≥2 biological replicates.

The effects of CORM-3 on cell outer membranes were assayed using the fluorescent probe *N*-phenyl-1-napthylamine (NPN) (42), a membrane-impermeable dye that has a weak fluorescence emission in buffer but increased fluorescence on exposure to a hydrophobic environment. Thus, when the bacterial membrane becomes perturbed (for example, by addition of an antibiotic or, here, CORM-3), the dye partitions into the outer membrane, leading to an increase in fluorescence. The potent respiratory inhibitor, potassium cyanide (KCN) was added, where indicated, to cell suspensions to prevent the expulsion of the dye by *E. coli* cells and give simpler fluorescence kinetics (12). CORM-3 perturbed the membrane of wild-type and *hemA E. coli* cells in the presence and absence of KCN (Fig. 8); a control with NPN incubated with cells alone showed no increase in basal fluorescence levels over 60 min. Since KCN is an inhibitor of terminal heme-mediated respiration, which is lacking in the *hemA* mutant, it was not surprising that KCN had less effect on the fluorescence profiles in *hemA* cells (compare Fig. 8E, G) than in wild-type cells (compare Fig. 8A, C). Interestingly, in mutant cell suspensions, basal levels of NPN fluorescence were greater than in the wild-type, reflected in the fluorescence intensity at zero time. Addition of CORM-3 to *hemA* cells, with or without KCN, led to higher fluorescence than in wild-type cells, suggesting greater damage to the membrane by CORM-3 perhaps due to an already compromised outer membrane. In wild-type cells, neither equimolar CO nor iCORM-3 significantly increased NPN fluorescence (Fig. 8B, D). However, in *hemA* cells, particularly in the presence of KCN, iCORM-3 but not CO elicited fluorescence increase, suggesting membrane destabilization (Fig. 8F, H).

We recently reported that CORM-3 has a role in K^+^/Na^+^ ion transport across the membrane of *E. coli* cells (74). To investigate whether this process was dependent on heme-containing proteins and correlated with membrane integrity, movement of K^+^/Na^+^ was explored by testing osmotic swelling of *hemA* spheroplasts in iso-osmotic solutions. Interestingly, no swelling was observed in response to CORM-3 in iso-osmotic solutions of K^+^ or Na^+^ salts (Supplementary Fig. S7) whereas wild-type spheroplasts swell with CORM-3 in these media (74). Controls with the K^+^ ionophore valinomycin and the metal cation (Supplementary Fig. S7A, B), and use of the nonionic detergent Triton X-100, confirmed the osmotic sensitivity of the spheroplasts (Supplementary Fig. S7). This probably reflects the membrane destabilization in *hemA* cells and loss of osmotic selectivity. The mechanisms of CORM-promoted ion transport in bacteria are not understood, but the present data indicate an energy- (respiration-) dependent transport mechanism in wild-type cells that is activated by CORM-3.

### Modeling of transcriptomic data

Transcriptomic data sets are exceptionally informative; however, their very wealth can occasionally make interpretation difficult. The measured altered patterns of gene expression shown in Figs. 3, 4, and 6 and Supplementary Figs. S5 and S6 pass a statistical filter, but it is beneficial to use further statistical methods to explain the changes in terms of more interpretable factors. In this study, we use the TFInfer approach (4, 65), a Bayesian statistical method that integrates gene expression data with regulon information (collected from online databases such as Regulon DB or Ecocyc) to identify transcription factor (TF) activity profiles that optimally explain the measured changes in gene expression.

**FIG. 9.**
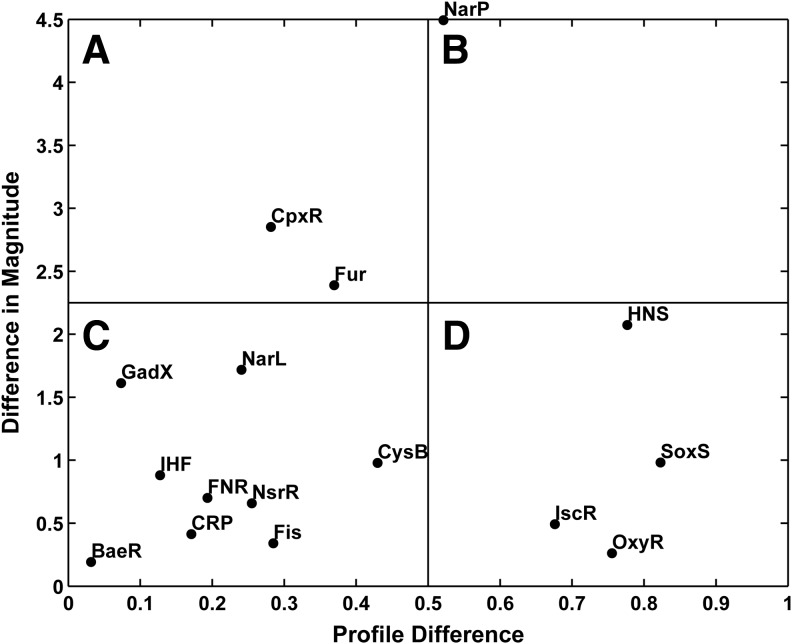
**Coherence plot showing transcription factors (TFs) involved in the response to CORM-3** ***versus*** **iCORM-3 in** ***hemA*** **cells.** The x-coordinate of each point represents the “profile difference” between the two conditions (computed as 1 minus the absolute Pearson correlation coefficient between the two profiles), while the y-coordinate represents the change in magnitude of the response (computed as the difference of the norm of the two profiles). Hence, TFs whose response is similar in both magnitude and kinetics will be located near the origin of the coherence plot in quadrant C, while TFs in quadrant B of the plot respond very differently both in terms of kinetics and in terms of amplitude. The activity of the TF BaeR in *hemA* cells is similar in response to both iCORM-3 and CORM-3, whereas HNS and NarP respond differently when cells are exposed to iCORM-3 or CORM-3.

We ran TFInfer separately on the CORM-3 and iCORM-3 data sets and devised an intuitive visualization method that highlights differences in the magnitude of the response, and differences in the kinetics of the response, to the two stimuli. Namely, for each TF, we plot on the abscissa the profile difference (computed as 1 minus the absolute Pearson correlation coefficient between the two profiles) *versus* the difference in magnitude of the response on the ordinate (computed as the absolute difference of the norms of the two profiles, Table 1 and Fig. 9). We term these plots *coherence plots*. Hence, TFs whose response is similar in both magnitude and kinetics will be located near the origin of the coherence plot, while TFs in the top right corner of the plot respond very differently in both kinetics and amplitude. Several of the regulators whose activity is inferred to underlie effects described in this paper feature in this analysis. Thus, CpxR, which appears in quadrant A of Figure 9, is a member of the two-component regulatory system CpxA/CpxR that combats extra-cytoplasmic protein-mediated toxicity by increasing the synthesis of the periplasmic protease DegP as well as that of CpxP protein. The position of CpxP in the matrix is consistent with the membrane disturbance elicited by CORM-3 but not iCORM-3 (Fig. 8). The response regulator BaeR, however, which confers resistance to novobiocin and bile salts by stimulating drug exporter gene expression is near the origin in the coherence plot (Fig. 9), indicating that its response is similar in terms of magnitude and kinetics when cells are exposed to iCORM-3 or CORM-3, and is not markedly upregulated (Fig. 7). In contrast, H-NS gave a low correlation coefficient for this comparison, indicating a specific response to CORM-3 (Fig. 9).

**Table 1.** Absolute Pearson Correlations Comparing Transcription Factors of Interest

	*hemA CORM-3 vs. iCORM-3*		*Wild type vs. hemA (CORM-3)*	
*Transcription factor name*	*Mean absolute Pearson correlation*	*Standard deviation of correlation*	*Mean absolute Pearson correlation*	*Standard deviation of correlation*
BaeR	0.968	0.017	0.810	0.043
CpxR	0.718	0.039	0.959	0.008
CRP	0.829	0.019	0.814	0.014
CysB	0.570	0.034	0.535	0.036
Fis	0.715	0.030	0.860	0.023
FNR	0.807	0.014	0.766	0.013
Fur	0.630	0.019	0.987	0.004
GadX	0.927	0.037	0.064	0.048
H-NS	0.223	0.043	0.859	0.024
IHF	0.873	0.015	0.862	0.014
IscR	0.324	0.055	0.812	0.036
NarL	0.759	0.052	0.382	0.246
NarP	0.479	0.249	0.386	0.240
NsrR	0.745	0.047	0.820	0.067
OxyR	0.244	0.089	0.889	0.037
SoxS	0.177	0.132	0.814	0.111

Mean absolute Pearson correlation values are given for transcription factor activity profiles in the heme-deficient mutant of *Escherichia coli* (*hemA*) treated with CORM-3 *versus* iCORM-3 and the heme-deficient mutant *versus* wild-type after exposure to CORM-3. Values close to 1 indicate transcription factors that exhibit a similar pattern of activity over 2 h post-treatment of heme-deficient or wild-type *E. coli* with the compound. Listed also is the resulting standard deviation of the absolute Pearson correlation.

These outcomes reflect the fold changes shown in Figure 7 and reveal the presence of targets for CORM-3 in *hemA* cells, although the molecular mechanisms underpinning the H-NS response remain uncertain. The coherence plot also reveals that OxyR and SoxS respond similarly in terms of magnitude when *hemA* cells are exposed to CORM-3 or iCORM-3, but the activity profiles are dissimilar. Interestingly, these two TFs were implicated in a transcriptomic profiling study of *E. coli* exposed to CORM-2 and subsequently mutated (51); deletion of these oxidative stress-sensing regulators increased CORM-2 sensitivity. In a final example, the TF IscR, for “iron-sulfur cluster regulator” (72), is negatively autoregulated, and it contains an Fe-S cluster that acts as a sensor required for components of a secondary pathway of cluster assembly and integration into Fe-S proteins, and respiratory enzymes. Thus, IscR responds similarly in terms of magnitude when *hemA* cells are exposed to CORM-3 or iCORM-3, but the activity profiles are dissimilar. Each point on the plot has associated horizontal and vertical error bars that take into account the uncertainty of the inferred TF activities derived from TFInfer. These error bars have been removed from Figure 9 to reduce visual clutter. The same plot with error bars is shown in Supplementary Fig. S8.

## Discussion

The idea that CO liberated from a CORM has targets other than heme has been proposed in light of the multifaceted CORM-induced alterations in the transcriptome of bacterial cells (51). However, the data presented here provide the first clear evidence that a CORM is toxic to bacteria in the absence of heme, a “classical” target of CO. Growth with CO gas did not inhibit growth of the heme-deficient mutant of *E. coli* and only marginally slowed wild-type growth (Supplementary Fig. S2A, B). The slight effects observed with iCORM-3 (Supplementary Figs. S3 and S4) are attributed to residual CO release from the compound (Supplementary Fig. S3A inset), and/or the low turbidity at which the cultures were stressed, as the inactive compound did not affect the viability of *L. lactis* after addition at mid-log phase of growth (Supplementary Fig. S4D). Collectively, the data show that a synergistic effect of CO and the ruthenium co-ligand fragment are required for full bactericidal activity.

The enhanced toxicity of CORM-3 in the absence of heme suggests that heme proteins protect the wild-type from the full potential of CORM-3 toxicity, perhaps, in part, by acting as a “CO-sink.” Furthermore, *hemA* cells accumulated more CORM-3 than wild-type cells (Fig. 2); metal accumulation presumably contributes to toxicity of the CORM. Reconstitution of the mutant's ability to synthesize heme by adding δ-ALA yielded cultures that were sensitive in the first 2 h to 200 μ*M* CORM-3, but later recovered to wild-type levels of viability (Fig. 1F). This may be explained by incomplete reconstitution, that is, the degree of heme synthesis was not fully restored to wild-type levels. Samples of wild-type cells grown under anaerobic conditions contained 0.68 nmol cytochrome *b* (mg protein)^−1^ and 0.53 nmol cytochrome *d* (mg protein)^−1^, in comparison with 0.46 and 0.36 nmol mg^−1^ in the reconstituted mutant, respectively.

Interestingly, the transcriptomic analyses highlight a profound effect of CORM-3, and to a lesser extent iCORM-3, on iron homeostasis even in the heme-deficient mutant (Fig. 5). In mammalian cells, disruption of heme synthesis elicits massive import of iron, presumably because the lack of iron is “sensed” by the cell as an indication of iron deficiency (23). Altered expression levels of some of these genes have been previously reported in wild-type cells, namely downregulation of *ftnA* and *bfr* and upregulation of *ftnB* (15, 51) under anaerobiosis. In this study, *hmp* is highly upregulated by CORM-3 in the *hemA*, but it is unaltered in the wild type (Fig. 7). The response may relate to the ferrisiderophore reductase activity of Hmp (3, 56), consistent with the iron-starvation response observed here. However, the ability of Hmp to reduce iron(III) *in vitro* is not believed to have physiological significance.

Statistical modeling of the transcriptomic data inferred the activity of FNR and Fur, which are involved in iron homeostasis. An interaction of CO with the iron centers of these TFs has been previously suggested (15). In its active form, Fur contains a nonheme ferrous iron site but, on iron deprivation, iron(II) is lost from the protein, resulting in de-repression of genes involved in siderophore biosynthesis and iron transport (61). Fur is also capable of sensing other metal ions, including zinc(II) (1), which may be relevant to the involvement of Fur in the response to CORM-3. Davidge *et al.* (15) suggested an interaction of CO with the [4Fe-4S] cluster of FNR, which reacts with NO (13). A gene implicated in the repair of damaged Fe-S clusters (*ytfE*) (35), and perhaps iron centers in general (54), was also upregulated in the mutant treated with CORM-3, and an interaction of CORM-2 with Fe-S clusters has recently been reported: CORM-2 lowers the activity of the Fe-S-containing aconitase and glutamate synthase (68).

In addition to CORM-3 effects on iron homeostasis, targeting of the cell membrane is indicated here as a major casualty of CORM-3 stress, by both CORM-enhanced NPN fluorescence assays of membrane integrity (Fig. 8) and upregulation of several genes implicated in stress responses at the membrane and in the periplasm. Here, we also show for the first time that CORM induced changes in bacterial protein levels, specifically proteins involved in the cell envelope response—Spy and CpxP. Upregulation of *spy* transcript levels (Fig. 6) and Spy protein (Fig. 7) can occur due to envelope stress (58); *cpxP* encodes the inhibitor of the Cpx response that is activated by misfolded envelope proteins (14, 59). These findings are consistent with previous transcriptomic studies (16, 48, 51), yet here we show that the effects are independent of hemes in the membrane. The TFs CpxR and BaeR identified by statistical modeling (Fig. 9) regulate the expression of *spy* and a number of other genes encoding membrane proteins. Both of these TFs may therefore have roles in the maintenance of envelope integrity and response to envelope stress after exposure of both the mutant and the wild type to CORM-3. Future work should extend the intensive transcriptomic and modeling approaches described here, already supported by immunoblotting of selected key proteins, by applying the concept of proteomic signatures (73) as a diagnostic tool to pinpoint the targets of CORM action.

## Materials and Methods

### Bacterial strains and growth conditions

Wild-type strains of *E. coli* K12 MG1655 and *L. lactis* (kind gift from Dr. Marc Solioz, University of Berne, Switzerland) were used. The heme-deficient mutant of *E. coli* (W3310 *hemA*::Km^R^) was obtained from the Keio collection (6, 75) and the mutant allele P1-transduced into strain MG1655. Cells were grown anaerobically in defined medium, pH 7, (24) supplemented with 0.1% casamino acids and 5% LB (15), 0.5% (w/v) glucose as carbon source, δ-ALA (final concentration 0.1 m*M*) where indicated, and kanamycin (final concentration 50 μg/ml) for the heme-deficient mutant.

For growth and viability assays, cells were grown at 37°C in 8 ml anaerobic tubes with screw-tops containing a Suba-Seal to maintain anaerobiosis during additions and sampling. Cultures were inoculated with 1% v/v of overnight cultures grown in rich broth (K_2_HPO_4_ (4 g/L), KH_2_PO_4_ (1 g/L), tryptone (10 g/L), and yeast extract (5 g/L), adjusted to pH 7).

For transcriptomic studies, *E. coli* were grown in batch cultures in custom-made, stirred (200 rpm) 250 ml mini-fermenter vessels at 37°C (40) during continuous sparging with nitrogen to maintain anaerobiosis. Cultures were inoculated with 5% v/v of cells grown overnight in rich broth that were harvested and resuspended in defined medium. OD measurements were made using a Jenway 7315 spectrophotometer.

For bacterial growth with CO gas, cells were incubated in defined medium in batch culture in an Infors Multifors bioreactor adapted to fit a Labfors-3 fermenter base unit. Temperature was 37°C with continuous stirring at 200 rpm, and mass flow controllers allowed the flow of gas at 100 ml min^−1^. Cultures were grown to an OD_600_ of ∼0.2 in pure nitrogen before switching to CO.

Under these conditions, cells grew at rates that approximated to linear, not exponential, kinetics. Therefore, growth rates are expressed as doubling times (h) at the point of adding CORMs or CO gas, not specific growth rates (h^−1^). The values given are means of 3 separate growths±SD.

### Analysis of cytochrome content of bacterial whole cells

Cultures were grown anaerobically in defined medium in 100 ml Duran bottles for 24 h (mutant cells) or 7–8 h (reconstituted cells) during gentle rotation on a roller mixer. Cells were harvested, washed in 0.1 *M* KPi (pH 7), and resuspended to give a thick suspension. Cytochromes were quantified using an SDB-4 dual-wavelength scanning spectrophotometer and dithionite-reduced *minus* persulfate-oxidized difference spectra obtained as earlier (36). For CO plus reduced *minus* reduced difference spectra, reduced samples were bubbled with CO for 2 min. Protein concentrations were measured using the Markwell assay (46). Concentrations of cytochromes *b* and *d* were calculated from reduced *minus* oxidized spectra using established extinction coefficients: *b* (560–575 nm; ɛ 17.5 m*M* cm^−1^) and *d* (630–655; ɛ 19 m*M* cm^−1^) (34).

### CORM-3 and control treatments

CORM-3 has been described earlier (11). iCORM-3 was prepared as described earlier (11, 41, 74) to give a preparation that releases to ferrous myoglobin typically <5% of the CO delivered by equimolar CORM-3. The basis for the inability of iCORM-3 to release CO is poorly understood, since CORM-3 does not release CO spontaneously (10). The compounds (74) were added directly to cultures of *E. coli* at an OD_600_ of 0.1–0.2, and *L. lactis* at an OD_600_ of 0.4.

### Bactericidal assays

Serial dilutions of culture samples between 10^−1^ and 10^−8^ were prepared in 1×PBS. From each dilution, 10 μl drops were plated onto rich broth agar, adjusted to pH 7, and incubated overnight at 37°C. The average number of colonies was calculated from the dilution giving the highest number of colonies without confluence and used to determine the number of colony-forming units per ml (CFU/ml).

### Metal analyses

Cultures were grown to log phase (for wild-type OD_600_ 0.4 and for *hemA* OD_600_ 0.2) where 20 ml samples were taken both before and after the addition of 100 μ*M* CORM-3. Samples were assayed for metal content as earlier (48).

### Microarray analysis

Microarray experiments were performed as earlier (48) except that, for RNA isolation, bacteria were grown and treated with CORM-3 in batch culture. In the microarray data, arbitrary values of ≥2-fold or ≤0.5-fold changes in expression were chosen to represent significantly altered genes. Information about gene products and their function was obtained from GeneSpring GX v7.3 (Agilent Technologies). Functional category lists were created using KEGG (Kyoto Encyclopedia of Genes and Genomes) (37). Relevant regulatory proteins for each gene were identified (where available) using regulonDB and EcoCyc (World Wide Web). The data have been deposited in NCBI's Gene Expression Omnibus and are accessible through GEO series accession number GSE55097 (www.ncbi.nlm.nih.gov/geo/query/acc.cgi?acc=GSE55097).

### Mathematical modeling of transcriptomic data

Modeling of TF activities using TFInfer (4, 65) and measuring similarity in TF activities between two different conditions were performed as described in (48).

### Western blotting for Spy and CpxP detection

Wild-type MG1655 and *hemA* cells were grown in *hemA* defined minimal medium as described earlier. When cultures reached the mid-exponential phase, CORM-3 was added to a final concentration of 20 μ*M* and incubated for 1, 2, or 4 h. Cells were harvested, and periplasmic fractions were isolated using the Tris/sucrose/EDTA (TSE) method (57). For Western blotting with anti-CpxP, soluble fractions were made by suspending cell pellets in 0.5 ml Tris-HCl buffer (pH 7.4) and sonication at 15 μm for two cycles of 30 s. Soluble fractions were isolated by ultracentrifugation at 30,000 rpm at 4°C for 30 min and reduced with 200 m*M* dithiothreitol before separation by SDS-PAGE on NuPAGE 4–12% Bis-Tris Gels (Life Technologies). Proteins were blotted onto Hybond-P polyvinylidene difluoride membrane (Amersham). Immunoblots were carried out using primary rabbit anti-Spy/CpxP antibodies at 1:25,000/1:50,000 dilutions, respectively. Anti-rabbit secondary antibodies were incubated at a concentration of 1:50,000 for 1 h before detection using the ECL-Plus Western blotting detection system (Amersham) with Hyperfilm ECL (Amersham).

### OM permeabilization assays

OM permeability of CORM-3 was assayed using NPN (32, 42). Cultures were grown to exponential phase (OD_600_ of 0.6 for wild type and 0.3 for *hemA*), pelleted, then washed, and resuspended in PBS. The final cell suspension was adjusted to an OD_600_ of ∼0.5. Cells were incubated with NPN (final concentration 1 μ*M*) and, where indicated, KCN (final concentration 1 m*M*). Fluorescence was measured (λ_ex_=340 nm, λ_em_=420 nm) using a Hitachi F-2500 fluorescence spectrophotometer.

### Spheroplasts and osmotic swelling measurements

*hemA* cells were grown anaerobically in LB supplemented with 20 m*M* glucose to an OD_600_ of ∼0.6, and spheroplasts were prepared (43). Cells were washed in 10 m*M* Tris-HCl (pH 7.4) and resuspended in 20% w/v sucrose containing 33 m*M* Tris-HCl (pH 8). Spheroplast formation was as described earlier (43) except that EDTA/lysozyme treatment was at 37°C (74). Osmotic swelling was measured by monitoring change in turbidity at 500 nm using a Cary 50 spectrophotometer (Varian) (27) after dilution of spheroplasts in iso-osmotic 0.25 *M* solutions of KNO_3_, KNO_2_, or NaNO_3._

## Supplementary Material

Supplemental data

Supplemental data

Supplemental data

Supplemental data

Supplemental data

Supplemental data

Supplemental data

Supplemental data

## Abbreviations Used

δ-ALA: δ-aminolevulinic acid
CFU: colony-forming units
CORM: carbon monoxide-releasing molecule
CORM-3: Ru(CO)_3_Cl(glycinate)
Fe-S: iron-sulfur (as in cluster)
iCORM-3: inactivated CORM-3
ICP-AES: inductively coupled plasma atomic emission spectroscopy
KCN: potassium cyanide
KPi: potassium phosphate buffer
Mb-CO: carboxymyoglobin
NPN: *N*-phenyl-1-naphthylamine
OD: optical density
OM: outer membrane
PBS: phosphate-buffered saline
TF: transcription factor
