# Supplementary material for: CO-Releasing Molecules Have Nonheme Targets in Bacteria: Transcriptomic, Mathematical Modeling and Biochemical Analyses of CORM-3 [Ru(CO)3Cl(glycinate)] Actions on a Heme-Deficient Mutant of Escherichia coli
Source: Antioxid Redox Signal. 2015 Jul 10;23(2):148–62. doi: 10.1089/ars.2014.6151 (PMC4492677; doi:10.1089/ars.2014.6151)
Supplement: Supplemental data [file Supp_Figure1.pdf]

Supplementary Data

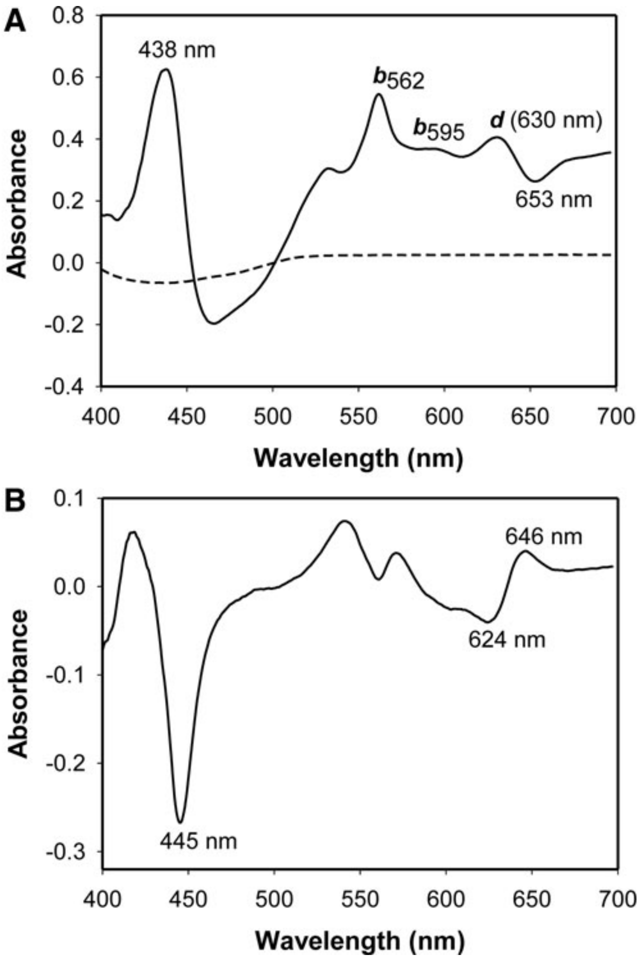

SUPPLEMENTARY FIG. 1. Difference spectra confirm the lack of cytochromes in the *hemA* mutant and their reconstitution. Cultures were grown as previously described (but without CORM-3 treatment) for 7–8 h (reconstituted cells) or 24 h (mutant cells), then harvested, washed thoroughly, and resuspended in 0.1 *M* KPi (pH 7). Cellular cytochrome content was assessed by dual wavelength spectrophotometry at room temperature. The resultant data were smoothed in SigmaPlot (Systat Software Inc.). Recorded wavelength positions are shown. **(A)** Reduced *minus* oxidized spectra of the *hemA* mutant (*dashed line*, 17.3 mg protein ml^{-1}) and after reconstitution with δ-ALA (*solid line*, 21.8 mg protein ml^{-1}). **(B)** CO-reduced *minus* reduced spectrum of cells after reconstitution with δ-ALA. Concentrations of cytochromes *b* (0.46 nmol mg protein^{-1}) and *d* (0.36 nmol mg protein^{-1}) were calculated from the reduced *minus* oxidized spectrum after reconstitution with δ-ALA.
