# Supplementary material for: CO-Releasing Molecules Have Nonheme Targets in Bacteria: Transcriptomic, Mathematical Modeling and Biochemical Analyses of CORM-3 [Ru(CO)3Cl(glycinate)] Actions on a Heme-Deficient Mutant of Escherichia coli
Source: Antioxid Redox Signal. 2015 Jul 10;23(2):148–62. doi: 10.1089/ars.2014.6151 (PMC4492677; doi:10.1089/ars.2014.6151)
Supplement: Supplemental data [file Supp_Figure2.pdf]

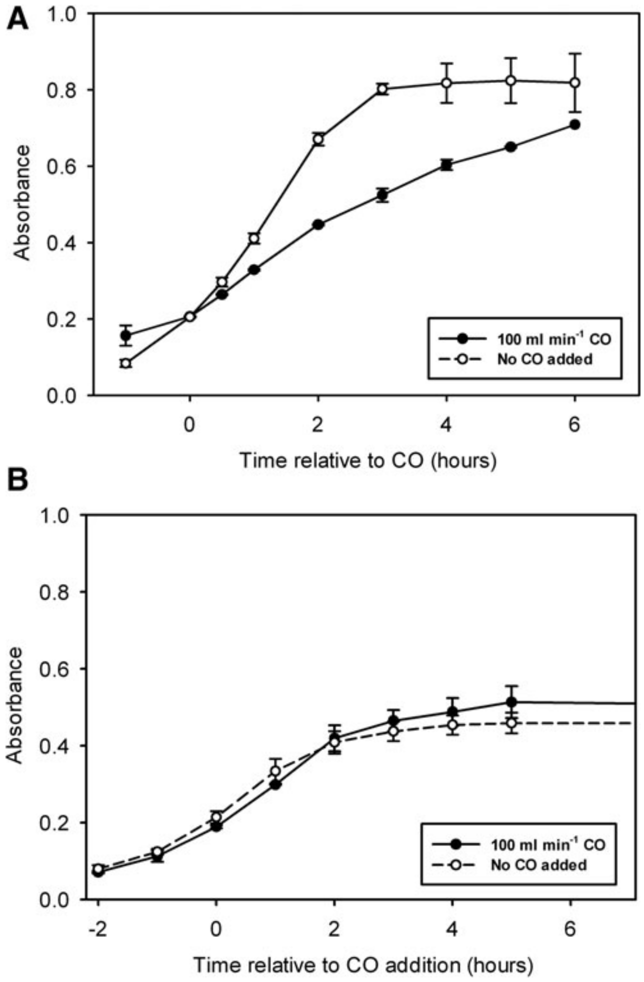

SUPPLEMENTARY FIG. 2. Growth of the heme-deficient mutant is unaffected by CO gas. Wild-type **(A)** and *hemA* **(B)** cultures were grown anaerobically in defined medium and bubbled with nitrogen gas at a rate of 100 ml min^{-1}. At an OD_{600} of ~ 0.2, the gas supply was switched to CO gas (*closed circles*, 100 ml min^{-1}) or maintained as nitrogen (*open circles*) and growth was monitored. Data are plotted as means $\pm$ SEM from two biological replicates.
