# Supplementary material for: CO-Releasing Molecules Have Nonheme Targets in Bacteria: Transcriptomic, Mathematical Modeling and Biochemical Analyses of CORM-3 [Ru(CO)3Cl(glycinate)] Actions on a Heme-Deficient Mutant of Escherichia coli
Source: Antioxid Redox Signal. 2015 Jul 10;23(2):148–62. doi: 10.1089/ars.2014.6151 (PMC4492677; doi:10.1089/ars.2014.6151)
Supplement: Supplemental data [file Supp_Figure3.pdf]

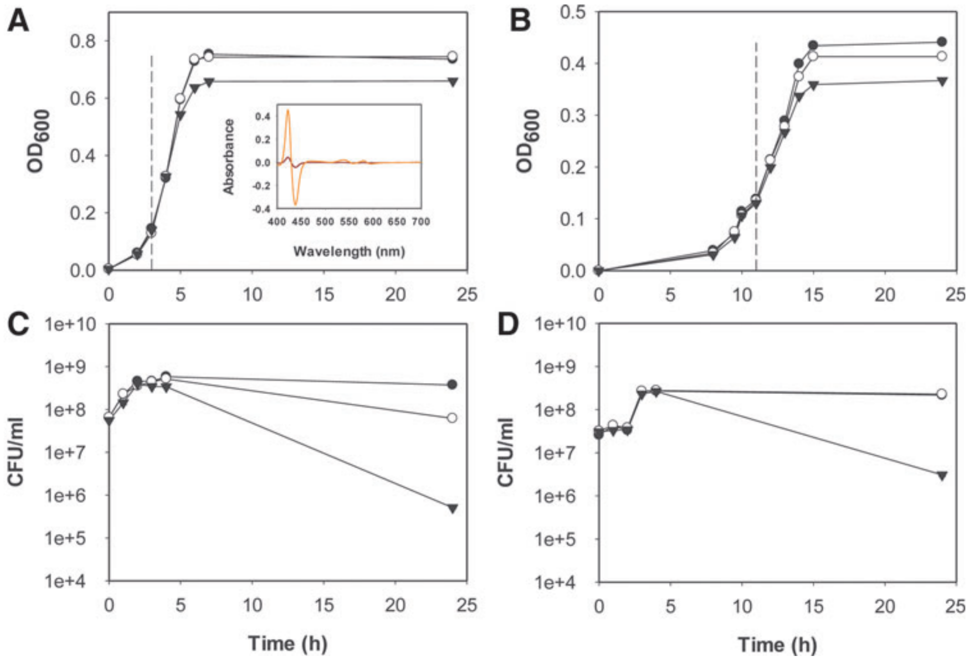

SUPPLEMENTARY FIG. 3. Effects of iCORM-3 on wild-type and heme-deficient *E. coli*. Cultures were grown as previously described and treated with 100 μM (*open circles*) and 200 μM (*closed triangles*) iCORM-3 at an OD_{600} of ~ 0.1 (*dashed lines*). Control cultures are shown in parallel (*closed circles*). Effects of iCORM-3 on wild type **(A)** and mutant **(B)** growth, and wild type **(C)** and mutant **(D)** viability are shown. Note that in D the points for the control culture (*closed circles*) lie beneath those for the 100 μM CORM-3 data (*open circles*). The *inset* in A shows CO difference of myoglobin tested with CORM-3 (*orange*) and iCORM-3 (*red*). For viability assays, a culture sample was taken immediately before addition of the inactive compound ($t = 0$ in **C and D**) followed by hourly samples for 4 h, and at 24 h to complete the experiment. Data represent the pattern seen in ≥ 3 biological replicates. Viability data are plotted as means $\pm$ SEM from ≥ 3 individual spots. Note that the scale on the y-axis is logarithmic in base 10, hence $1e+3 = 1000$.
