# Supplementary material for: CO-Releasing Molecules Have Nonheme Targets in Bacteria: Transcriptomic, Mathematical Modeling and Biochemical Analyses of CORM-3 [Ru(CO)3Cl(glycinate)] Actions on a Heme-Deficient Mutant of Escherichia coli
Source: Antioxid Redox Signal. 2015 Jul 10;23(2):148–62. doi: 10.1089/ars.2014.6151 (PMC4492677; doi:10.1089/ars.2014.6151)
Supplement: Supplemental data [file Supp_Figure4.pdf]

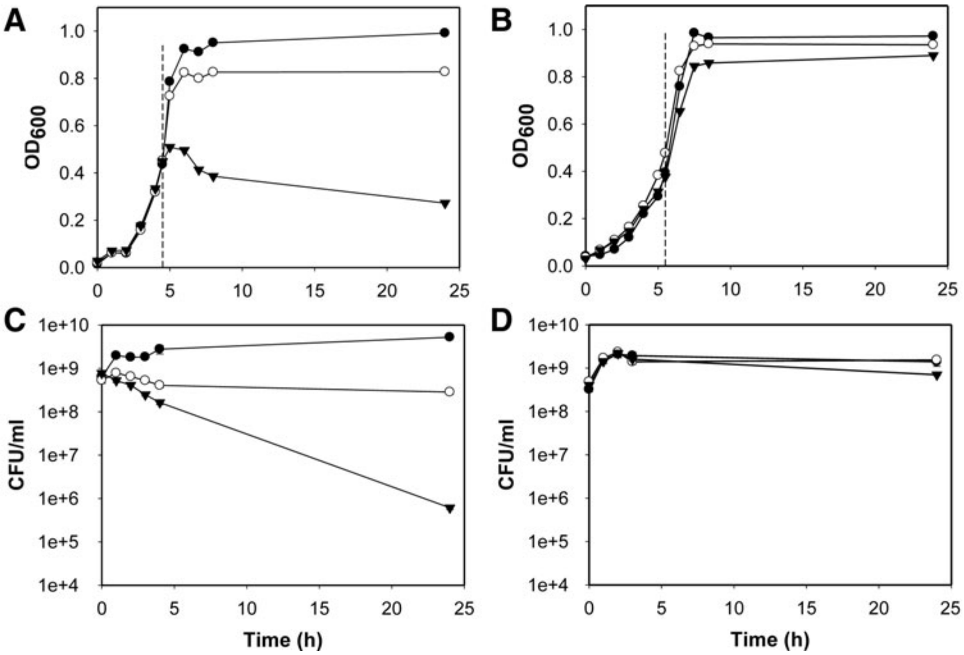

SUPPLEMENTARY FIG. 4. CORM-3, but not iCORM-3, is bactericidal against naturally heme-deficient *Lactococcus lactis*. Cultures were grown anaerobically in defined medium and stressed with 100 μM (*open circles*) and 200 μM (*closed triangles*) of compound at an OD_{600} of ~ 0.4 (*dashed line*). The effect of CORM-3 on growth **(A)** and viability **(C)**, and the effect of iCORM-3 on growth **(B)** and viability **(D)** are shown. Control cultures are shown in parallel (nothing added, *closed circles*). A culture sample was taken immediately before compound addition to assay for cell viability ($t = 0$ in **B and D**). Hourly OD_{600} readings and culture samples were taken for 3–4 h postaddition of compound, followed by a 24 h reading to complete the experiment. Data represent the pattern seen in ≥ 3 biological replicates. Viability data are plotted as means $\pm$ SEM from three individual spots. Note that the scale on the y-axis is logarithmic in base 10, hence $1e + 3 = 1000$.
