# Supplementary material for: CO-Releasing Molecules Have Nonheme Targets in Bacteria: Transcriptomic, Mathematical Modeling and Biochemical Analyses of CORM-3 [Ru(CO)3Cl(glycinate)] Actions on a Heme-Deficient Mutant of Escherichia coli
Source: Antioxid Redox Signal. 2015 Jul 10;23(2):148–62. doi: 10.1089/ars.2014.6151 (PMC4492677; doi:10.1089/ars.2014.6151)
Supplement: Supplemental data [file Supp_Figure5.pdf]

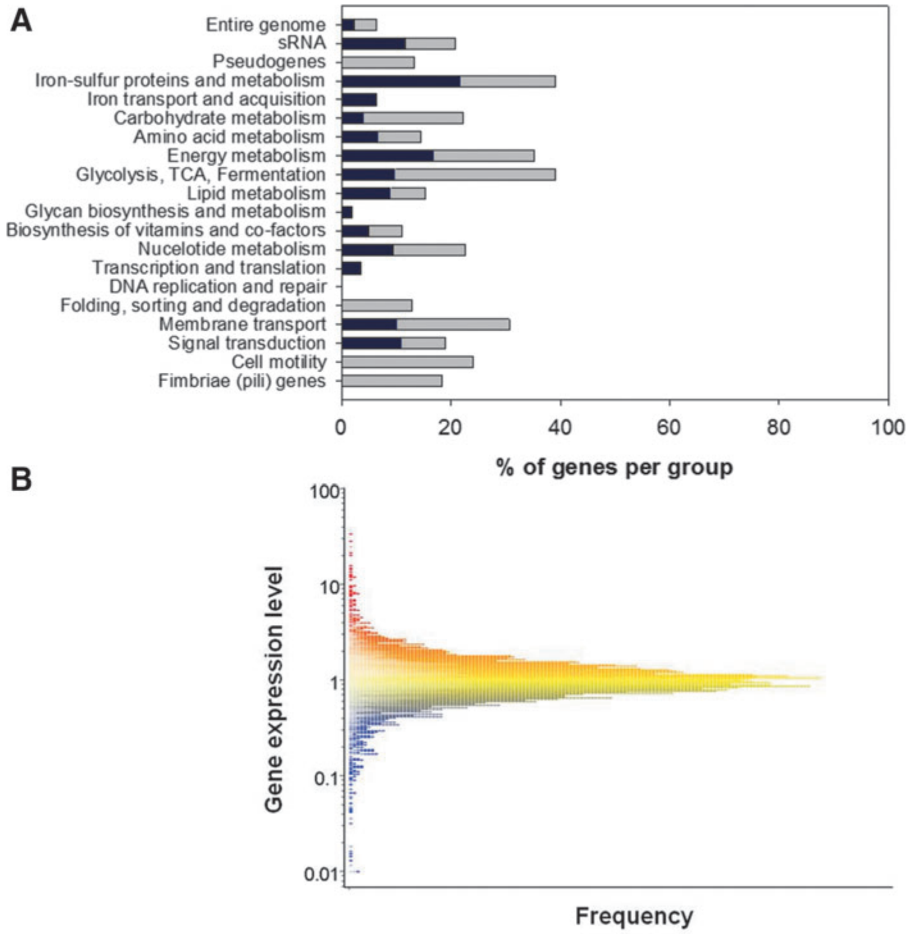

SUPPLEMENTARY FIG. 5. Differences in gene expression between the heme-deficient mutant and wild type immediately before addition of CORM-3. (A). The bars show the percentage of genes in functional groups with altered expression in the heme-deficient mutant *versus* wild type. The dark *blue* and *grey bars* indicate the proportion of up- and downregulated genes, respectively. **(B).** Expression profile showing the frequency of genes with altered transcription in the heme-deficient mutant *versus* wild type. *Red* indicates upregulated genes ($\geq$2-fold), *blue* indicates downregulated genes ($\geq -$ 2- fold), and *yellow* represents genes with unaltered expression.
