# Supplementary material for: CO-Releasing Molecules Have Nonheme Targets in Bacteria: Transcriptomic, Mathematical Modeling and Biochemical Analyses of CORM-3 [Ru(CO)3Cl(glycinate)] Actions on a Heme-Deficient Mutant of Escherichia coli
Source: Antioxid Redox Signal. 2015 Jul 10;23(2):148–62. doi: 10.1089/ars.2014.6151 (PMC4492677; doi:10.1089/ars.2014.6151)
Supplement: Supplemental data [file Supp_Figure6.pdf]

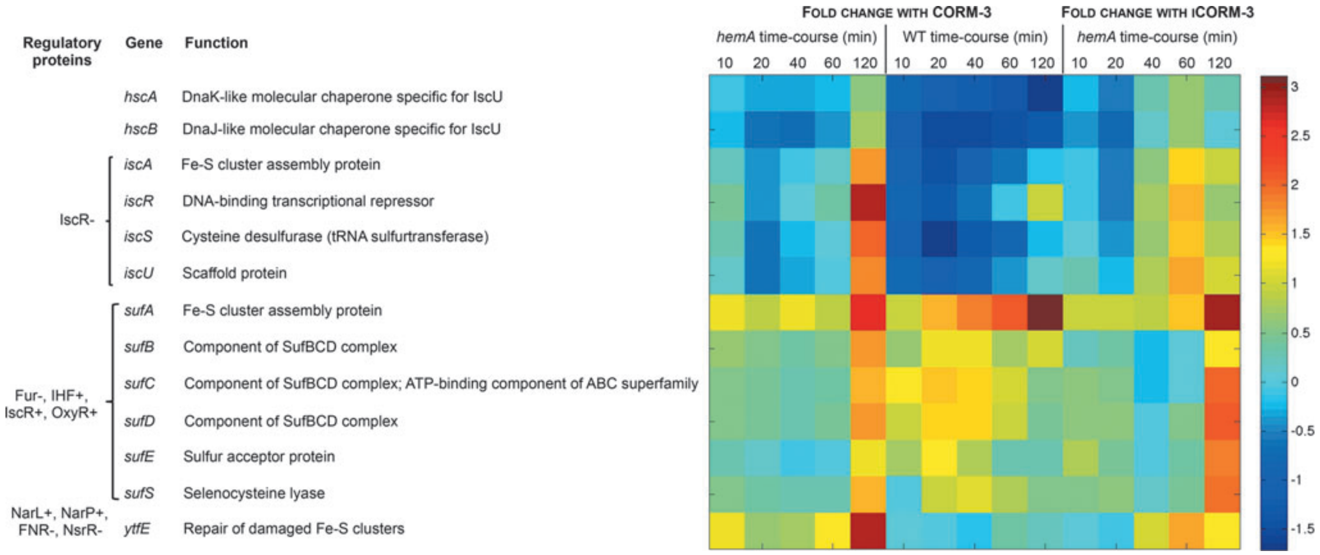

SUPPLEMENTARY FIG. 6. Differential expression of genes involved in Fe-S cluster assembly and repair. The heat map quantifies the changes in selected genes; it should be noted that the ''heat scale'' at the *right* is expressed as the natural logarithm of the fold change in genes in the heme-deficient mutant of *E. coli* (*hemA*) and the corresponding wild type grown anaerobically in defined medium after addition of 100 μM CORM-3 or, for the mutant only, 100 μM iCORM-3.
