# Supplementary material for: CO-Releasing Molecules Have Nonheme Targets in Bacteria: Transcriptomic, Mathematical Modeling and Biochemical Analyses of CORM-3 [Ru(CO)3Cl(glycinate)] Actions on a Heme-Deficient Mutant of Escherichia coli
Source: Antioxid Redox Signal. 2015 Jul 10;23(2):148–62. doi: 10.1089/ars.2014.6151 (PMC4492677; doi:10.1089/ars.2014.6151)
Supplement: Supplemental data [file Supp_Figure7.pdf]

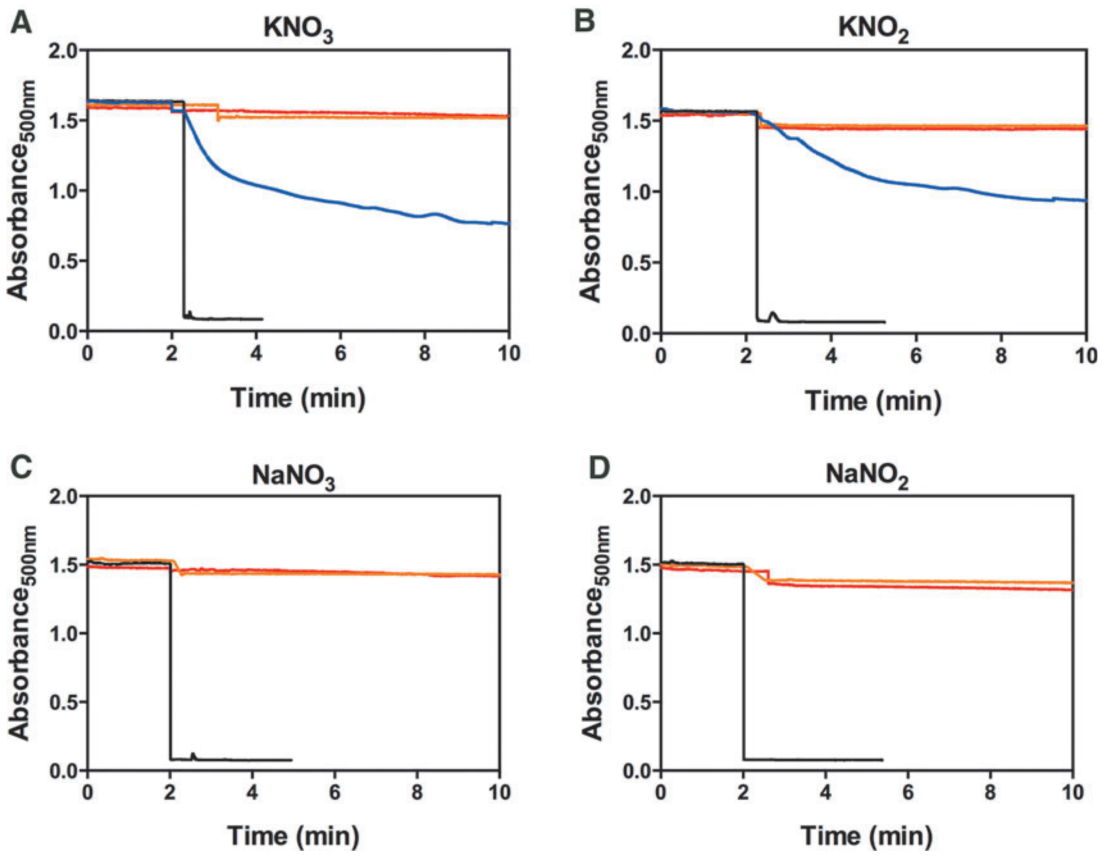

SUPPLEMENTARY FIG. 7. CORM-3 does not promote K^+ and Na^+ fluxes across the membrane of *hemA* cells. Osmotic behavior of *hemA* spheroplast suspensions (OD_{500nm} of ~1.5) in **(A)** 0.25 *M* potassium nitrate (KNO_3), **(B)** 0.25 *M* potassium nitrite (KNO_2), **(C)** 0.25 *M* sodium nitrate ($NaNO_3$), or **(D)** 0.25 *M* sodium nitrite (KNO_2) with additions of Triton-X-100 (*black line*), valinomycin (*blue line*), CO gas (*orange line*), or 200 μM CORM-3 (*red line*). Data are representative of three biological replicates.
