# Supplementary material for: CO-Releasing Molecules Have Nonheme Targets in Bacteria: Transcriptomic, Mathematical Modeling and Biochemical Analyses of CORM-3 [Ru(CO)3Cl(glycinate)] Actions on a Heme-Deficient Mutant of Escherichia coli
Source: Antioxid Redox Signal. 2015 Jul 10;23(2):148–62. doi: 10.1089/ars.2014.6151 (PMC4492677; doi:10.1089/ars.2014.6151)
Supplement: Supplemental data [file Supp_Figure8.pdf]

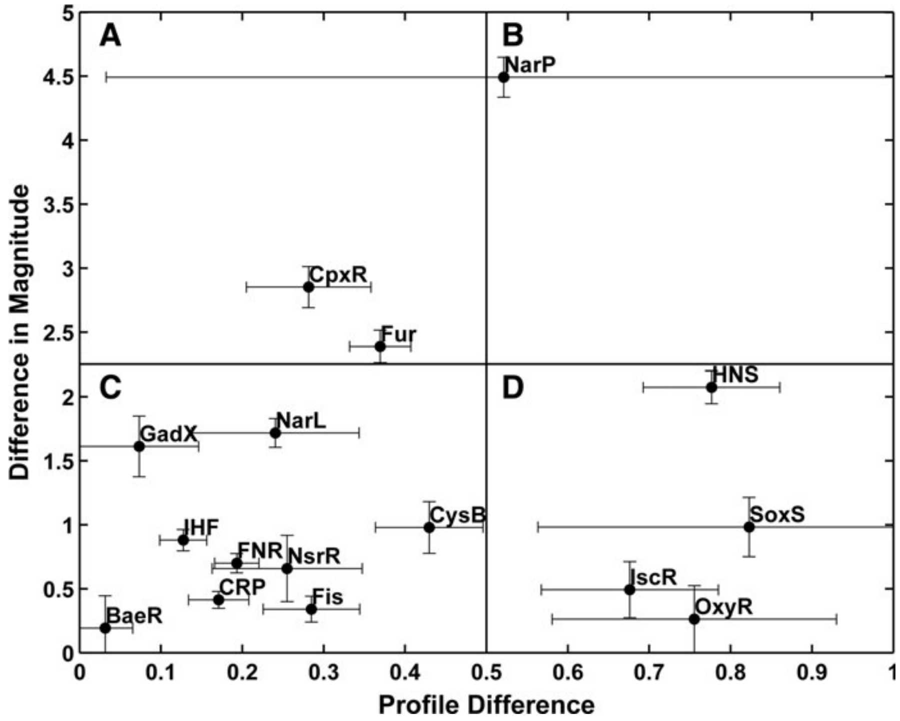

SUPPLEMENTARY FIG. 8. Coherence plot showing TFs involved in the response to CORM-3 *versus* iCORM-3 in *hemA* cells. The same data as in Fig. 9. are shown here with error bars (1.96 times standard deviation, or 95% confidence interval) for the profile difference and difference in magnitude, to give an idea of the uncertainty in these measurements. The uncertainty in this case arises from the uncertainty of the inferred transcription factor profiles derived from TFInfer (4, 65). Quadrants A–D have the same meaning as in Fig. 9 and as described in the text.
